# Evaluation of Body Composition in Hemodialysis Thai Patients: Comparison between Two Models of Bioelectrical Impedance Analyzer and Dual-Energy X-Ray Absorptiometry

**DOI:** 10.1155/2018/4537623

**Published:** 2018-08-05

**Authors:** Kulapong Jayanama, Supanee Putadechakun, Praopilad Srisuwarn, Sakda Arj-Ong Vallibhakara, Prapimporn Chattranukulchai Shantavasinkul, Chanika Sritara, Surasak Kantachuvesiri, Surat Komindr

**Affiliations:** ^1^Chakri Naruebodindra Medical Institute, Faculty of Medicine Ramathibodi Hospital, Mahidol University, 270 Rama 6 Road Thungphayathai, Ratchathewi, Bangkok 10400, Thailand; ^2^Division of Nutrition and Biochemical Medicine, Department of Medicine, Faculty of Medicine Ramathibodi Hospital, Mahidol University, 270 Rama 6 Road Thungphayathai, Ratchathewi, Bangkok 10400, Thailand; ^3^Section for Clinical Epidemiology and Biostatistics, Faculty of Medicine Ramathibodi Hospital, Mahidol University, 270 Rama 6 Road Thungphayathai, Ratchathewi, Bangkok 10400, Thailand; ^4^Division of Nuclear Medicine, Department of Radiology, Faculty of Medicine Ramathibodi Hospital, Mahidol University, 270 Rama 6 Road Thungphayathai, Ratchathewi, Bangkok 10400, Thailand; ^5^Division of Nephrology, Department of Medicine, Faculty of Medicine Ramathibodi Hospital, Mahidol University, 270 Rama 6 Road Thungphayathai, Ratchathewi, Bangkok 10400, Thailand

## Abstract

**Background:**

Body composition measurement is very important for early nutritional care in hemodialysis patients. Dual-energy X-ray absorptiometry (DXA) is a gold standard test, but clinically limited. Bioelectrical impedance analysis (BIA) with multifrequency technique is a practical and reliable tool.

**Objective:**

This cross-sectional study was aimed to compare the agreement of BIA with DXA in measurement of body composition in hemodialysis patients and to evaluate their associated factors.

**Methods:**

Body composition was measured by 2 BIA methods (InBody S10 and InBody 720) and DXA after a hemodialysis session. A total of 69 measurements were included. Pearson's correlation and Bland and Altman analysis were used to determine the correlation of body composition between methods and to compare the methods agreement, respectively.

**Results:**

The correlation coefficients of body compositions were strong between DXA and InBody S10 (fat mass index (FMI): *r*=0.95, fat-free mass index (FFMI): *r*=0.78) and also between DXA and InBody 720 (FMI: *r*=0.96, FFMI: *r*=0.81). Comparing to DXA, the means of each body composition measured by InBody S10 method were not significantly different in each gender, but differences were found in FM, %FM, and FMI measured by InBody 720.

**Conclusions:**

In maintenance hemodialysis patients, the measurement of body composition with DXA and both BIA methods had highly significant correlations; practically, BIA method could be used as an instrument to follow FM and FFM and to measure the edematous stage. Further studies with large populations are warranted.

## 1. Introduction

The prevalence of chronic kidney disease (CKD) in the Thai population becomes much higher than previously known [1]. As a consequence, the patients undergoing renal replacement therapy of hemodialysis have been rising [2]. Regular hemodialysis causes the decrease of both FM and FFM over times which is independently associated with a higher mortality and a tendency toward a worse quality of life [3]. These also increase the prevalence of protein-energy malnutrition [4] and morbidity [5].

Early detection of malnourishment and optimized nutritional care can improve the outcomes [6]. Therefore, the body composition analysis is one of the most important strategies to assess and monitor the nutritional status. Searching for a practical and accurate tool for body composition evaluation is essential.

Dual-energy X-ray absorptiometry (DXA), based on the signals from two energy sources to provide a three compartment model of body composition, is taken place and has become a gold standard test [7]. DXA is a reproducible and reliable technique for measuring fat mass in healthy [8] as well as in hemodialysis patients [9]. Unfortunately, this costly device, which is nonportable and depended on proficiency, cannot be used as a practical or accessible bedside tool. Additionally, the body compartments, in particular total body water (TBW) in chronic hemodialysis population, are significantly altered comparing to healthy population [10]. Bioelectrical impedance analysis (BIA) with multifrequency technique has been proven to be one of the most valid methods comparing to DXA with high correlation in the healthy population [11]. However, the estimation by the BIA analyzers compared with that measured by DXA in hemodialysis patients was found slightly higher in fat mass (FM) and slightly lower in fat-free mass (FFM), but significantly [12]. The error of the BIA was found greater in patients with CKD than in healthy subjects [13]. Because of its readily accessible, low cost, and quickly assessing procedure, the BIA method has become widely used in clinical practice, in sport medicine, and also in weight reduction programs [7, 14].

To date, none of the studies have investigated the agreement between BIA and DXA in measurement body composition in hemodialysis Thai population. Due to the effect of race [15], the accuracy of BIA must be evaluated. The aims of the present study were to compare the agreement between BIA and DXA in measurement of the body composition and to evaluate their associated factors.

## 2. Materials and Methods

### 2.1. Subjects

This study was a cross-sectional study conducted in the hemodialysis unit of Ramathibodi Hospital, Mahidol University, Bangkok, Thailand. All subjects, aged more than 18 years, who had regularly been on maintenance hemodialysis for at least 3 months prior to the study were included. The study was performed during October 2013 and May 2014. Patients were treated with three sessions of dialysis per week on schedule, lasting 4 hours per period. The percentage of renal replacement therapy methods was found 53% with hemodialysis and 47% with hemodiafiltration. This study excluded all subjects who were unstable or currently on medications affecting metabolic rate or admitted in the hospital during the period of study. All of the measurements were done on the dialysis day. Baseline data including age, sex, nutritional information, education background, socioeconomic status, physical examinations, and anthropometric measurements were performed and completed by the well-trained physician.

The protocol was approved by the Institutional Review Board, Faculty of Medicine, Ramathibodi Hospital, Mahidol University (Approval number MURA2013/317 Ns_1_ Feb_17_). Written informed consent was obtained from each participant.

### 2.2. Measurement and Laboratory Determinations

Anthropometric parameters including weight, height, and waist circumference were measured twice with standard techniques by the same skillful physician. All measurements were performed in the same day after the subjects had fasted for 12 hours and within 30 minutes after termination of hemodialysis period. All participants dressed in light clothes without shoes. Body mass index (BMI) was calculated by the postdialysis body weight (kilogram (kg)) divided by the height squared (meter (m)^2^).

BIAs were measured by 2 models of multifrequency impedance analyzers (model InBody S10, Biospace Co., Ltd., Seoul, Korea, and model InBody 720 Biospace Co., Ltd., Seoul, Korea) which provide 6 different frequency impedance measurements (1, 5, 50, 250, 500, and 1000 kHz) and 3 different frequencies of phase angle measurement (5, 50, and 250 kHz) at each 5 segments (right arm, left arm, trunk, right leg, and left leg). Model InBody S10 was conducted while the patient was lying supine for 15 minutes on a bed with legs apart and arms not touching the torso after all metals were removed. The touch type electrodes were placed following the manuscript of the model, whereas model InBody 720 was performed while the patient was standing upright: hands hold the electrodes and feet on the electrodes, with 8-point tactile electrode method. The output values included the intracellular fluid, extracellular fluid, FM, FFM, and %FM. By extracellular fluid (ECF)/(TBW), edematous state was detected by the BIA method [16]. In addition, the FM index (FMI) was determined by the postdialysis FM (kg) divided by the height squared (m^2^), and FFM index (FFMI) was determined by the postdialysis FFM (kg) divided by the height squared (m^2^).

DXA was performed, immediately after BIA measurement in the same day, using the Hologic Discovery A instrument (Hologic Inc., Waltham, MA, USA). All scans were performed by the same trained technician and analyzed by the same radiologist. The calibration was done each day prior to start of testing by the standard technique. The assessed data were FM, FFM, %FM, and bone mass.

MIS (Malnutrition-Inflammation Score) is the scoring system more comprehensive and quantitative evaluation criteria composed of 10 components: the 7 Dialysis Malnutrition Score components and 3 new items (body mass index (BMI), serum albumin level, and total iron-binding capacity (TIBC)) have been added. In a recent prospective study, the MIS was found to be a comprehensive scoring system which had the significant associations with prospective hospitalization and mortality [17, 18].

### 2.3. Statistical Methods

All statistical analyses were performed using STATA 12.0 software (StataCorp. 2011: Stata Statistical Software: Release 12. College Station, TX: StataCorp LP). Mean ± standard deviation (SD) or median (interquartile range (IQR)) for continuous variable and frequency (%) for binary or categorical variable were presented. Paired *t*-tests were used to compare mean of FM, %FM, FFM, FMI, and FFMI measured by BIA and DXA. The correlation between FM, %FM, FFM, FMI, and FFMI predicted by BIA and those measured by DXA was determined by Pearson's correlation coefficient (*r*). The Bland and Altman analysis [19] was used to compare the agreement between the measurement techniques. The limits of agreement between methods were defined as the mean difference ±1.96 SD (95% limits of agreement). The correlation between the intermethod differences and each body parameter was obtained by Pearson's correlation coefficient (*r*) test. A statistical significance was attained when a *p* value was less than 0.05.

## 3. Results

Sixty-nine measurements were performed. A total of 66% of patients were male, and the mean age was 59.66 ± 11.28 years, ranging from 40 to 87 years. Patient BMIs were between 17.41 and 35.76 kg/m^2^: 19% of them had a BMI of <18.5 kg/m^2^, and 47% had a BMI of >23 kg/m^2^. By MIS, 64% of patients were defined as severe malnutrition. Edematous state was detected by both BIA methods (ECF/TBW). The averages of normalized protein catabolic rate (nPCR), Kt/v, and dialysis vintage were 0.91 ± 0.20 g/kg, 1.78 ± 0.39, and 5.21 (2.22–12.74) years, respectively (Table 1).

In both genders, no significant intermethod difference of FM, %FM, FFM, FMI, and FFMI measured by InBody S10 and DXA was found; on the other hand, means of FM, %FM, and FMI measured by BIA 720 were significantly higher than when measured by BIA S10 and DXA (Table 2). The significant difference in means of FM, %FM, and FMI measured by all 3 methods was not observed between genders. Nevertheless, all methods showed significantly higher FFM and FFMI in men (*p* < 0.001).

**Figure 1:**
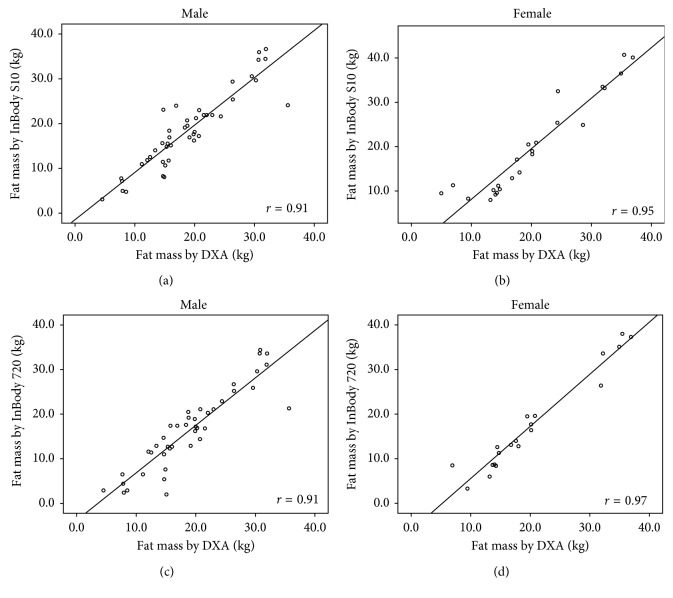
Correlation between fat mass in hemodialysis patients: (a) measured by DXA and BIA S10 in male, (b) measured by DXA and BIA S10 in female, (c) measured by DXA and BIA 720 in male, and (d) measured by DXA and BIA 720 in female.

**Figure 2:**
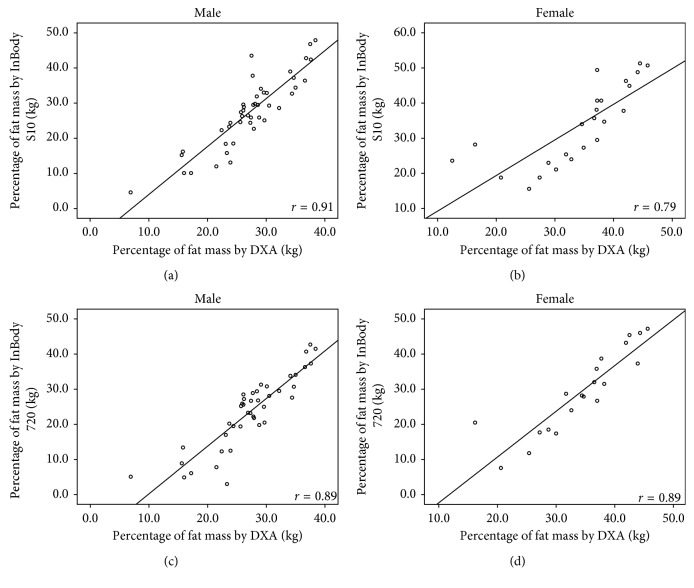
Correlation between percentages of fat mass in hemodialysis patients: (a) measured by DXA and BIA S10 in male, (b) measured by DXA and BIA S10 in female, (c) measured by DXA and BIA 720 in male, and (d) measured by DXA and BIA 720 in female.

**Figure 3:**
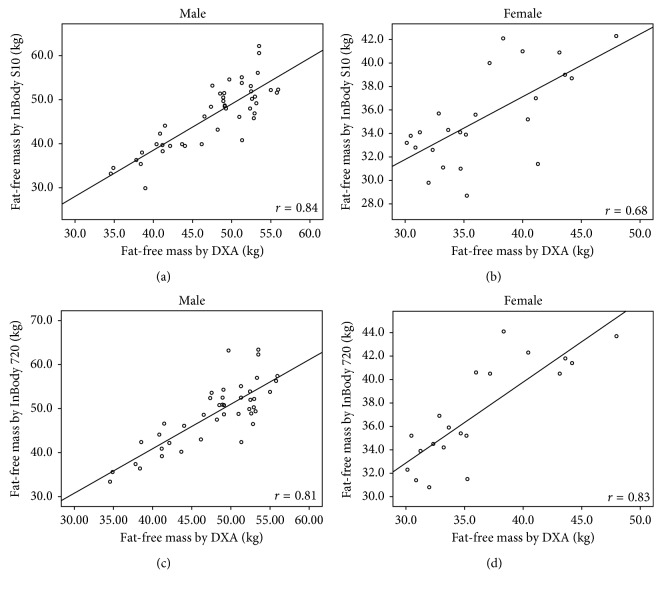
Correlation between fat-free mass in hemodialysis patients: (a) measured by DXA and BIA S10 in male, (b) measured by DXA and BIA S10 in female, (c) measured by DXA and BIA 720 in male, and (d) measured by DXA and BIA 720 in female.

**Figure 4:**
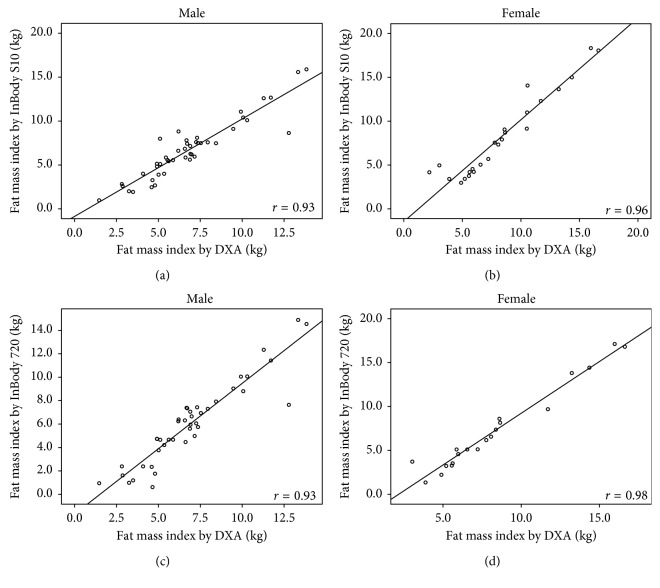
Correlation between fat mass index in hemodialysis patients: (a) measured by DXA and BIA S10 in male, (b) measured by DXA and BIA S10 in female, (c) measured by DXA and BIA 720 in male, and (d) measured by DXA and BIA 720 in female.

**Figure 5:**
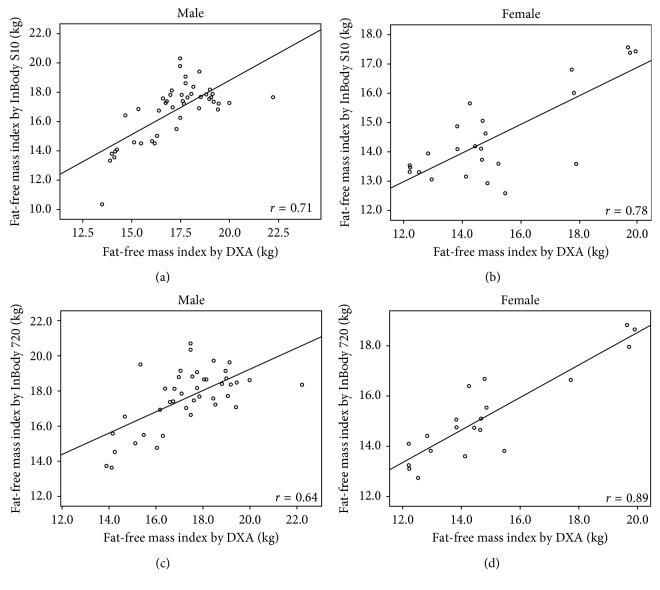
Correlation between fat-free mass index in hemodialysis patients: (a) measured by DXA and BIA S10 in male, (b) measured by DXA and BIA S10 in female, (c) measured by DXA and BIA 720 in male, and (d) measured by DXA and BIA 720 in female.

The correlation coefficients (*r*) of body compositions between DXA and InBody S10 were high (FM: *r*=0.93; %FM: *r*=0.85; FFM: *r*=0.88; FMI: *r*=0.95; FFMI: *r*=0.78) with *p* < 0.001. Strong correlations were also found between DXA and InBody 720 (FM: *r*=0.94; %FM: *r*=0.88; FFM: *r*=0.89; FMI: *r*=0.96; FFMI: *r*=0.81) with *p* < 0.001. The correlation coefficients of these measurements between DXA and both BIA methods in each gender are illustrated in Figures 1–5.

**Figure 6:**
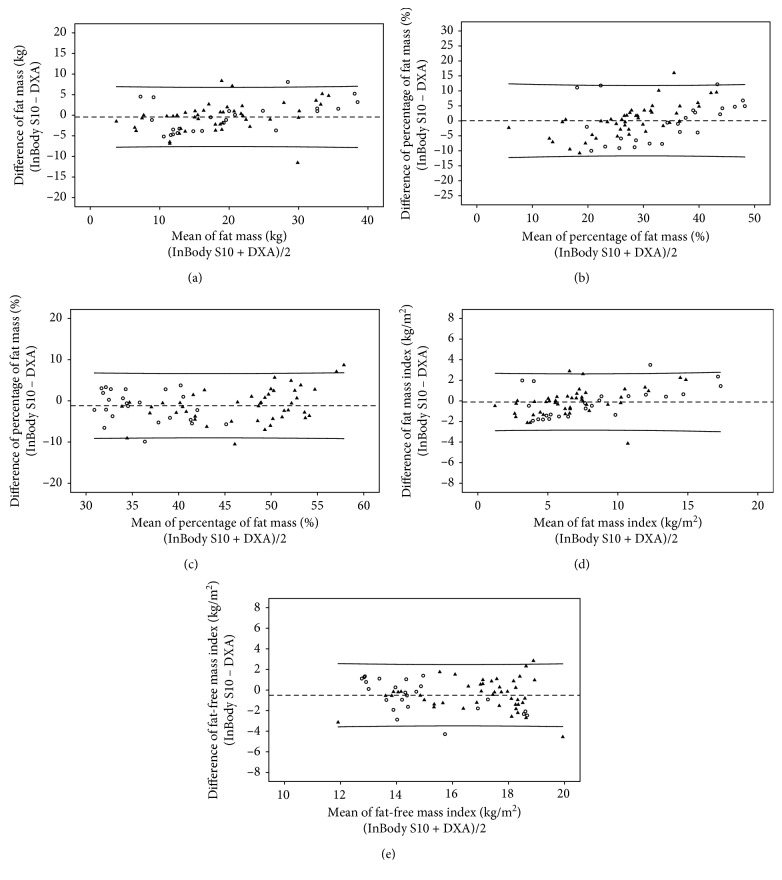
The agreement between DXA and BIA S10 in (a) fat mass, (b) percentage of fat mass, (c) fat-free mass, (d) fat mass index, and (e) fat-free mass index. Black filled-in triangles represent values of male, and black circles represent values of female.

**Figure 7:**
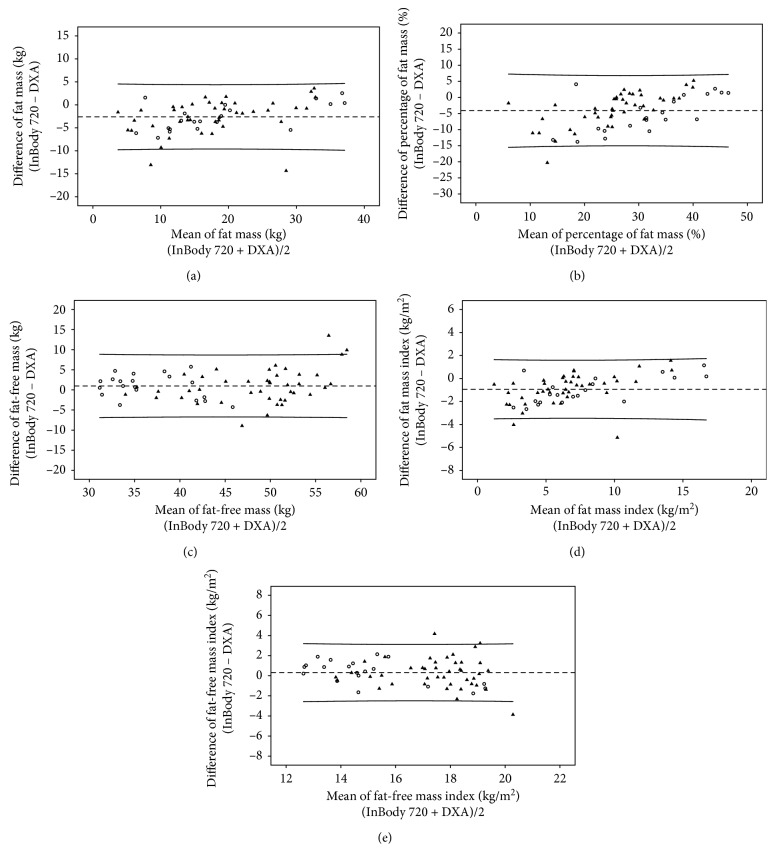
The agreement between DXA and BIA 720 in (a) fat mass, (b) percentage of fat mass, (c) fat-free mass, (d) fat mass index, and (e) fat-free mass index. Black filled-in triangles represent values of male, and black circles represent values of female.

By Bland and Altman analysis [19], the differences of FM, %FM, and FMI between DXA and InBody S10 method were smaller than that between DXA and InBody 720. However, both BIA methods had wide 95% limits of agreement with DXA (Table 3). These intermethod differences did not significantly differ between male and female. The agreements of all measures in both genders illustrated by Bland and Altman plots also revealed the same direction: between BIA S10 and DXA (Figure 6) and between BIA 720 and DXA (Figure 7). With regard to the measures between BIA S10 and DXA, the differences of %FM, FFM, and FFMI were significantly correlated with age (%FM: *r*=0.36, *p*=0.002; FFM: *r*=−0.34, *p*=0.004; FFMI: *r*=−0.35, *p*=0.003), body weight (%FM: *r*=0.25, *p*=0.039; FFM: *r*=−0.26, *p*=0.034; FFMI: *r*=−0.27, *p*=0.025), and edematous stage (%FM: *r*=0.39, *p*=0.001; FFM: *r*=−0.32, *p*=0.008; FFMI: *r*=−0.32, *p*=0.008), whereas the differences of FM and FFM were associated with only age (FM: *r*=0.40, *p*=0.001; FMI: *r*=−0.39, *p*=0.001) and edematous stage (FM: *r*=0.39, *p*=0.001; FMI: *r*=0.38, *p*=0.001). Nonetheless, any relationship between age and edematous stage and the difference measured by BIA 720 and DXA was not observed. Only body weight was found to have a significant association with the difference of % FM (*r*=0.39, *p*=0.002), FFM (*r*=−0.26, *p*=0.038), FMI (*r*=0.27, *p*=0.034), and FFMI (*r*=−0.31, *p*=0.016).

## 4. Discussion

The deterioration of body compositions, both FM and FFM, is strongly correlated with morbidity and mortality and represents a poor prognostic marker [20]. Hence, early nutritional care is very important to prevent this morbidity. Nevertheless, the measurement of FFM, which is predominantly composed of muscle mass, body water, and minerals, is affected by abnormal fluid and electrolyte distribution, and commonly observed in patients undergoing renal replacement therapy.

In agreement with the previous reports [7, 12], the present study observed highly significant correlations in the measurement of FM and FFM between DXA and both BIA methods. The FM measured by both BIA methods was lower than the DXA, and this could be the result of edema in hemodialysis patients in this study. The FFM of both genders was underestimated by InBody S10 but overestimated by InBody 720. As a result of gravity, the water distribution in the supine position differs from the upright position in the edematous state, [21] and the variation of body water distribution by BIA measurement of body composition is also affected by different positions [22]. All methods showed significantly higher FFM in men according to the normal physiology. The mean differences of FM, %FM, and FMI between DXA and InBody S10 method for both genders were small and not significant, whereas the mean differences between DXA and InBody 720 were higher. These results support the idea that the measurement of FM, %FM, and FFM by both BIA methods could be clinically, practically, and reasonably used in follow-up. BIA can also measure the edematous stage in this population. However, these devices cannot provide the accuracy of DXA when measuring FM and FFM.

The results between body compositions (FM and FFM) and their calculated indices (FMI and FFMI) measured by both BIA and DXA were in the same direction in this study. FMI and FFMI may be beneficial for nutritional assessment and easier recognition [23], but FM and FFM are also useful and familiar due to the direct report from the devices. The limitation of this study is the modest sample size. Nevertheless, this is the first study which compares DXA and both of 2 BIA methods in hemodialysis Thai patients.

## 5. Conclusions

In conclusion, the present study depicted that body composition values measured by DXA and both BIA methods had strongly significant correlations. However, significant differences between measurement by DXA and InBody 720 methods were also found but not between measurement by DXA and InBody S10. As a result, both BIA methods could be practically used as an instrument to follow FM, FFM, FMI, and FFMI in the same individuals. The body weight was an associated factor with the difference of FFM and FFMI when measured by DXA and both BIA methods. Notwithstanding, age and edematous stage were correlated with the intermethod difference when measured by DXA and InBody S10. Further study with a larger number of hemodialysis Thai patients should be warranted.

## Figures and Tables

**Table 1:** Demographic, anthropometric, and nutritional status parameters of the hemodialysis patients (mean ± SD).

Characteristics	Hemodialysis patients (mean ± SD)
Male, *n* (%)	24 (66)
Age (years)	59.15 ± 10.67
BW (kg)	61.51 ± 12.40
BMI (kg/m^2^), median (IQR)	22.65 (19.31–25.90)
BMI > 23 kg/m^2^, *n* (%)	17 (47)
Waist circumference (cm)	88.90 ± 12.81
Mid-upper arm circumference (cm)	28.13 ± 4.10
Triceps skinfold thickness, mm, median (IQR)	13.60 (11.45–19.20)
MIS	
A, *n* (%)	2 (5)
B, *n* (%)	11 (31)
C, *n* (%)	23 (64)
BIA (InBody S10)	
ECF (kg)	12.26 ± 2.17
ICF (kg)	19.10 ± 4.06
ECF/TBW	0.39 ± 0.02
BIA (InBody 720)	
ECF (kg)	13.67 ± 2.68
ICF (kg)	21.28 ± 3.95
ECF/TBW	0.39 ± 0.02
nPCR (g/kg)	0.91 ± 0.20
Kt/V	1.78 ± 0.39
Dialysis vintage (years), median (IQR)	5.21 (2.22–12.74)

BIA = bioelectrical impedance analysis; BW = body weight; ECF = extracellular fluid; ICF = intracellular fluid; IQR = interquartile range; MIS = Malnutrition-Inflammation Score; SD = standard deviation; TBW = total body water; nPCR = normalized protein catabolic rate.

**Table 2:** Body composition parameters of the hemodialysis patients with BIA S10, BIA 720, and DXA (mean ± SD).

	DXA	BIA S10	BIA 720
Fat mass (kg)			
Male	19.28 ± 7.30^a^	19.16 ± 8.29^b^	16.68 ± 9.07^a,b^
Female	20.33 ± 8.94^a^	19.88 ± 11.32^b^	16.40 ± 11.28^a,b^
Total	19.32 ± 7.94^a^	18.91 ± 9.36^b^	16.82 ± 9.46^a,b^

Percentage of fat mass (%)			
Male	27.24 ± 6.48^a^	27.53 ± 10.00^b^	23.68 ± 10.20^a,b^
Female	34.11 ± 8.70^a^	33.68 ± 11.22^b^	29.30 ± 11.57^a,b^
Total	29.63 ± 7.98^a^	29.67 ± 10.77^b^	25.49 ± 10.90^a,b^

Fat-free mass (kg)			
Male	47.56 ± 5.88	46.46 ± 7.35^a^	48.88 ± 7.24^a^
Female	36.66 ± 5.01	35.35 ± 3.96^a^	37.11 ± 4.35^a^
Total	43.77 ± 7.63^c,d^	42.6 ± 8.29^a,c^	45.08 ± 8.48^a,d^

Fat mass index (kg/m^2^)			
Male	6.83 ± 2.80^a^	6.72 ± 3.35^b^	6.05 ± 3.45^a,b^
Female	8.37 ± 3.91^a^	8.27 ± 4.75^b^	7.30 ± 4.77^a,b^
Total	7.37 ± 3.29^a^	7.26 ± 3.93^b^	6.46 ± 3.93^a,b^

Fat-free mass index (kg/m^2^)			
Male	17.18 ± 1.88	16.73 ± 1.95^a^	17.63 ± 1.70^a^
Female	15.10 ± 2.42	14.50 ± 1.52^a^	15.19 ± 1.80^a^
Total	16.46 ± 2.29^e^	15.95 ± 2.10^a,e^	16.84 ± 2.07^a^

BIA = bioelectrical impedance analysis; DXA = dual-energy X-ray absorptiometry; ^a,b^significant difference between tools of measurement at *p* < 0.001 in each line; ^c^significant difference between tools of measurement at *p*=0.014 in each line; ^d^significant difference between tools of measurement at *p*=0.049 in each line; ^e^significant difference between tools of measurement at *p*=0.006 in each line.

**Table 3:** Mean differences and limits of agreement for fat mass, percentage of fat mass, fat-free mass, fat mass index, and fat-free mass index of the hemodialysis patients with BIA S10, BIA 720, and DXA.

Methods	InBody S10 versus DXA		InBody 720 versus DXA	
	Mean difference ± SD	95% limits of agreement	Mean difference ± SD	95% limits of agreement
Fat mass (kg)				
Male	−0.42 ± 3.56	−7.40 to 6.56	−2.57 ± 3.74	−9.90 to 4.76
Female	−0.41 ± 3.67	−7.60 to 6.78	−2.70 ± 2.92	−8.42 to 3.02
Total	−0.41 ± 3.57	−7.42 to 6.59	−2.6 ± 3.47	−9.41 to 4.19

Percentage of fat mass (%)				
Male	0.28 ± 5.24	−9.99 to 10.55	−3.62 ± 5.24	−13.89 to 6.65
Female	−0.42 ± 6.93	−14.00 to 13.16	−5.19 ± 5.74	−16.44 to 6.06
Total	0.04 ± 5.84	−11.41 to 11.48	−4.13 ± 5.41	−14.73 to 6.48

Fat-free mass (kg)				
Male	−1.10 ± 4.00	−8.94 to 6.74	0.98 ± 4.21	−7.27 to 9.23
Female	−1.32 ± 3.74	−8.65 to 6.01	0.96 ± 2.93	−4.84 to 6.76
Total	−1.17 ± 3.88	−8.79 to 6.44	0.98 ± 3.82	−6.51 to 8.46

Fat mass index (kg/m^2^)				
Male	−0.11 ± 1.28	−2.62 to 2.40	−0.88 ± 1.30	−3.43 to 1.67
Female	−0.10 ± 1.52	−3.08 to 2.88	−1.06 ± 1.16	−3.33 to 1.21
Total	−0.11 ± 1.36	−2.78 to 2.56	−0.94 ± 1.25	−3.39 to 1.51

Fat-free mass index (kg/m^2^)				
Male	−0.46 ± 1.45	−3.30 to 2.38	0.29 ± 1.49	−2.63 to 3.21
Female	−0.60 ± 1.56	−3.66 to 2.46	0.35 ± 1.19	−1.98 to 3.52
Total	−0.51 ± 1.48	−3.41 to 2.39	0.31 ± 1.39	−2.41 to 3.03

BIA = bioelectrical impedance analysis; DXA = dual-energy X-ray absorptiometry.

## Data Availability

The datasets used and/or analyzed during the current study are available from the corresponding author on reasonable request.

## Abbreviations

BIA: Bioelectrical impedance analysis
BMI: Body mass index
BW: Body weight
CKD: Chronic kidney disease
DXA: Dual-energy X-ray absorptiometry
ECF: Extracellular fluid
FFM: Fat-free mass
FFMI: Fat-free mass index
FMI: Fat mass index
FM: Fat mass
ICF: Intracellular fluid
IQR: Interquartile range
kg: Kilogram
m: Meter
MIS: Malnutrition-Inflammation Score
nPCR: Normalized protein catabolic rate
SD: Standard deviation
TBW: Total body water
TIBC: Total iron-binding capacity.
